# A Longitudinal Study Examining the Impact of Chronic Rhinosinusitis on the Risk of Cancer Development: A National Population-Based Cohort Study

**DOI:** 10.3390/cancers17030546

**Published:** 2025-02-06

**Authors:** Dong-Kyu Kim, Jae-In Kim, Il Hwan Lee, Dae-Soon Son

**Affiliations:** 1 Department of Otorhinolaryngology-Head and Neck Surgery, Chuncheon Sacred Heart Hospital, Hallym University College of Medicine, Chuncheon 24252, Republic of Korea; 2 Department of Otorhinolaryngology-Head and Neck Surgery, OneSleep Clinic, Gunpo-si 15856, Republic of Korea; 3 Department of Physiology, Neurology, Hallym University College of Medicine, Chuncheon 24252, Republic of Korea; 4 Department of Data Science, Data Science Convergence Research Center, Hallym University, Chuncheon 24252, Republic of Korea

**Keywords:** rhinosinusitis, cancer, incidence, risk, cohort study

## Abstract

Background/Objectives: We investigated the association between chronic rhinosinusitis (CRS) and cancer risk in an adult Korean population. Methods: This retrospective cohort study used data from the Korean National Health Insurance Service database. To ensure comparability between the groups, adjustments were made for potential confounding factors, including sex, age, residence, household income, diabetes, hypertension, and chronic kidney disease. The primary endpoint was the presence of newly diagnosed cancer. Results: Among 1,337,120 individuals in the nationally representative cohort database, 10,567 patients with CRS were identified and matched with 42,268 control subjects without CRS. Patients with CRS had a significantly higher risk of overall cancer events than controls. The adjusted hazard ratio (HR) for cancer in the CRS group was 1.16 (95% confidence interval [CI]: 1.05–1.28). Notably, female patients with CRS had an elevated risk of incident cancer events. Furthermore, patients with CRS without nasal polyps exhibited a significantly increased risk of cancer, whereas those with CRS with nasal polyps did not show a similar association. Conclusions: These findings underscore the need for physicians to carefully monitor patients with CRS for potential cancer progression and develop appropriate therapeutic strategies to mitigate the impact of this condition.

## Simple Summary

Previous epidemiological studies have shown that chronic rhinosinusitis (CRS) is associated with an increased risk of various types of cancer and cancer-related mortality. Therefore, this study analyzed the association between CRS and cancer risk using a representative nationwide cohort database. Among 10,567 patients with CRS and 42,268 matched controls, CRS was linked to a higher cancer risk (adjusted HR: 1.16, 95% CI: 1.05–1.28). Female patients with CRS without nasal polyps exhibited a particularly high cancer risk. These findings highlight the need for careful monitoring and the development of tailored therapeutic strategies for CRS management.

## 1. Introduction

Chronic rhinosinusitis (CRS) is a persistent inflammatory disorder involving the nasal cavities and paranasal sinuses that lasts for more than 12 weeks despite appropriate medical management. It is characterized by symptoms such as nasal obstruction, congestion, or discharge, often accompanied by facial pain and/or a diminished sense of smell [1]. It is one of the most common chronic upper airway diseases diagnosed in otorhinolaryngology clinics. Its prevalence varies across studies and populations, but it is commonly estimated to affect approximately 5–12% of the general population [2,3]. Currently, CRS is clinically divided into two distinguishable phenotypes based on nasal endoscopic findings: CRS without nasal polyposis (CRSsNP) and CRS with nasal polyposis (CRSwNP). CRSwNP is a more severe form of CRS, and aspirin-exacerbated respiratory disease is frequently observed in patients with CRSwNP [4]. To date, various risk factors have been identified for the pathogenesis and progression of CRS, with some being shared across all CRS subtypes, whereas others are more specifically associated with particular forms of CRS. Several studies have highlighted that genetic factors, comorbid conditions such as airway diseases, gastroesophageal reflux disease, inflammatory and autoimmune disorders, and various demographic and environmental factors are linked to the development of CRS [3,5,6,7].

CRS is closely linked to persistent infection, dysfunction of the innate immune system, and chronic inflammation, all of which are thought to play crucial roles in the potential mechanisms driving tumorigenesis. The continuous cycle of inflammation and infection characteristic of CRS can lead to substantial changes in the tissue lining of the sinuses, which may disrupt the normal cellular environment and promote malignant transformation. The chronic inflammatory state in patients with CRS is believed to create a microenvironment conducive to tumor development, where inflammatory mediators, immune cell infiltration, and tissue remodeling processes can increase the risk of malignancy [8,9,10,11,12]. A previous study from Taiwan demonstrated that patients with CRS exhibit a notably higher cancer risk than those without CRS, with an increased incidence of cancers in the head and neck, breast, lung, bladder, colorectum, liver, prostate, and skin [9]. This study also showed that surgical treatment for CRS does not appear to reduce the elevated cancer risk [9]. Another study in Taiwan showed that CRS is associated with an increased risk of lung cancer, with a predisposition toward the adenocarcinoma subtype [12]. Although a prior study in South Korea demonstrated that patients with CRS have a significantly higher risk of developing specific head and neck cancers, including sinonasal, hypopharyngeal-laryngeal, and thyroid cancers, compared to individuals without CRS [8], no cohort study has yet investigated the association between CRS and the risk of developing all types of cancer in the Korean population. This highlights a critical gap in understanding the broader cancer burden associated with CRS. Thus, this study aims to address this gap by utilizing a matched cohort design to comprehensively evaluate the potential increased risk of all cancer types among patients with CRS.

Therefore, in this study, we investigated whether the risk of all cancer types increased using a propensity-matching analysis method that controlled for several confounding variables. By utilizing this large dataset, our study offers a unique opportunity to assess cancer risk in patients with CRS over an extended follow-up period, providing insights into the long-term health outcomes of patients with CRS. The inclusion of both patients with CRS and a control group allowed for a direct comparison, controlling for various confounding factors and ensuring the validity of the findings. The size and scope of the cohort also enhanced the generalizability of the results, making it one of the most extensive studies in this area to date. By analyzing a nationwide cohort, we aimed to better understand the relationship between CRS and cancer, an area that has been relatively underexplored in previous studies.

## 2. Materials and Methods

### 2.1. Representative Cohort Data from the National Health Claims Database

The establishment of a comprehensive national health insurance system in South Korea in 1989 facilitated universal access to healthcare for all citizens. Consequently, all residents are mandatorily registered with the Korea National Health Insurance Service (NHIS), which maintains centralized, systematically archived healthcare data. This centralized repository encompasses extensive medical records, including diagnostic codes, treatments, prescription information, and comprehensive demographic data, and effectively integrates patient-level information across the healthcare continuum. The data infrastructure is governed by strict regulatory oversight, ensuring systematic monitoring and allocation of medical resources among beneficiaries, healthcare providers, and governmental authorities. The dataset adheres to the International Classification of Diseases, 10th Revision, Clinical Modification (ICD-10-CM) for diagnostic coding, ensuring precision and consistency in the classification of disease states. Each member of the cohort was assigned a unique birth identifier that guaranteed the elimination of duplicate claims and prevented data omission, thereby maintaining the integrity and reliability of the data. The representativeness of this dataset allowed for an accurate depiction of the entire adult population in South Korea over a defined timeframe. This robust characteristic significantly mitigates potential selection bias and facilitates comprehensive research on health service usage, patient outcomes, and healthcare patterns. The longitudinal nature of this cohort provides an exceptional opportunity for researchers to conduct extended observational studies, enabling the assessment of temporal changes in health conditions, treatment pathways, and healthcare consumption. This capability is essential for identifying longitudinal trends, understanding healthcare behaviors, and projecting future healthcare demands. The comprehensiveness of this dataset makes it an invaluable asset for a wide array of epidemiological investigations, health policy assessments, and the formulation of public health strategies. This aids in analyzing disease incidence, treatment outcomes, and patient adherence to medical recommendations, thus contributing substantially to the optimization of healthcare services in South Korea.

### 2.2. Ethical Considerations and Data Accessibility

This study employed a nationally representative cohort dataset spanning 2002 to 2013, sourced from the South Korean National Health Claims Database provided by the NHIS. This dataset includes comprehensive healthcare information for 1,025,340 adults, constituting approximately 2.2% of the South Korean population. The large sample size and minimal data attrition of the cohort underscore its reliability for longitudinal analyses. Institutional approval for the study was obtained from the Institutional Review Board (IRB) of Hallym University Chuncheon Sacred Hospital (IRB No. 2021-08-006). Owing to the anonymized nature of the secondary data, the IRB waived the requirement for informed consent. In accordance with the data-sharing policies mandated by the Korea National Health Insurance Corporation, public access to the full dataset is restricted to safeguard patient privacy. However, pertinent data supporting the conclusions of this study have been incorporated into the analysis of the manuscript. Requests for additional data may be subject to approval from relevant institutional bodies and adherence to data protection regulations.

### 2.3. Retrospective Cohort Study Design and Statistical Analysis

The study population comprised individuals diagnosed with CRS during the index period from 2002 to 2004. CRS cases were identified using International Classification of Diseases, Tenth Revision, Clinical Modification codes J32 (CRS without nasal polyps, CRSsNP) and J33 (CRS with nasal polyps, CRSwNP). To qualify for inclusion, patients had to exhibit these diagnostic codes on two or more occasions within the index period or be hospitalized for a CRS diagnosis. CRS was defined as symptom persistence for at least 12 weeks. We excluded patients younger than 55 years, those who died during the index period, and those with a pre-existing cancer diagnosis. The control cohort was selected using propensity score matching at a ratio of four non-CRS controls per CRS patient to ensure comparability between the groups. The outcomes assessed in this study included all-cause mortality and cancer incidence. The cancer group was defined by the presence of identical C codes recorded more than three times within a single year or a C code used for inpatient admission, with diagnostic validation processes in place to confirm the diagnosis beyond insurance-related provisional coding. The primary endpoint of the study was cancer incidence, which was monitored until the end of the cohort period. Patients without recorded cancer diagnoses at the conclusion of follow-up were censored at that point. To control for potential confounders, data on patient age, sex, residence, household income, and pre-existing comorbidities were extracted from the database. Age groups were stratified into 55–69 years and ≥70 years. Income levels were classified as low (≤30% of the national median), middle (30.1%–69.9% of the national median), and high (≥70% of the national median). Residential regions were categorized as Seoul (the largest metropolitan area), other metropolitan cities, and smaller cities/rural areas. Comorbid conditions such as hypertension (I10), diabetes mellitus (E10–E14), and chronic kidney disease (N18) were identified using diagnostic records from 2003 to 2005.

To evaluate cancer incidence and compare risk ratios between patients with CRS and matched controls, we calculated cancer incidence per 1000 person-years from the point of enrollment to the study endpoints. Multivariable-adjusted logistic regression models, Cox proportional hazard models, and propensity score adjustments were used to account for potential confounding variables. The incidence rate was defined as the occurrence of disease or events per unit time (person-years) and was calculated from the initial CRS diagnosis until death, occurrence of a specific event, or the end of the study period for non-events. To estimate the hazard ratio (HR) and corresponding 95% confidence interval (CI), Cox proportional hazards regression analyses were conducted with adjustments for the identified confounding factors. Statistical analysis was performed using R software (version 3.5.0; R Foundation for Statistical Computing, Vienna, Austria), and a *p*-value of <0.05 was set as the threshold for statistical significance, ensuring robust inferential results.

## 3. Results

### 3.1. Cohort Sample Characteristics

In this study, two cohort sample datasets were extracted for analysis, comprising a total of 42,268 patients diagnosed with CRS and 10,567 patients without CRS. A comprehensive comparative analysis was conducted to assess the baseline characteristics of the two groups. The results revealed no significant differences in the baseline characteristics across the independent variables included in this study. This finding indicates that the two groups were well matched in terms of the variables under consideration, ensuring the reliability and validity of the comparison. The detailed matching of these variables is presented in Table 1, which further supports the adequacy of the cohort selection process for this study.

### 3.2. Analysis of the Incidence Rate and the Risk Ratio of Cancer in Patients with Chronic Rhinitis

Table 2 provides a detailed summary of the incidence rates and risk ratios for overall cancer events, offering a comprehensive analysis of the association between CRS and cancer development over a 12-year follow-up period. The analysis of incidence rates was based on a total of 12,382 person-years observed in individuals without CRS and 3279.1 person-years observed in patients diagnosed with CRS. These longitudinal data allowed for a robust comparison between the two groups in terms of cancer occurrence over time. Throughout the 12-year follow-up period, the overall incidence of all cancer events was 0.147 per 1000 person-years in individuals without CRS compared to 0.167 per 1000 person-years in those with CRS. This difference, although modest, suggests a trend toward a higher incidence of cancer in patients with CRS, highlighting the potential long-term cancer risk associated with this condition. To further assess the strength of this association, both univariate and multivariate Cox proportional hazards regression models were used to evaluate the HRs of cancer events in the two groups. The results of the multivariate analysis revealed that the CRS group exhibited a significantly higher risk of developing all types of cancer, with an adjusted HR of 1.16 (95% CI = 1.05–1.28). This adjusted HR indicated that patients with CRS were 16% more likely to develop cancer than those without CRS, even after accounting for potential confounders. These findings emphasize the increased cancer risk in patients with CRS, suggesting that CRS may be an important factor in cancer development. These results also underscore the need for ongoing monitoring and early detection strategies in patients with CRS to mitigate this elevated cancer risk.

### 3.3. Risk Analysis of Overall Cancer Events by Sex and Comorbidities

We performed a detailed risk analysis of overall cancer events, stratifying the data by sex to better understand the sex-specific variations in cancer risk associated with CRS. The results of this stratified analysis (Table 3) revealed a significant finding in female patients with CRS. Specifically, female patients with CRS were found to have a notably higher risk of developing cancer across all types of cancer events compared to female individuals without CRS, with an adjusted HR of 1.24 (95% CI: 1.10–1.41). This indicates that women with CRS are 24% more likely to develop cancer than their counterparts without the condition, after adjusting for potential confounders. In contrast, no significant difference in cancer risk was observed in male patients with CRS compared to their male counterparts without the condition. This lack of a statistically significant difference in cancer risk between male patients with CRS and non-CRS males suggests that the relationship between CRS and cancer development may differ between the sexes, with women being at a higher relative risk. These findings underscore the importance of considering sex-specific factors when assessing the long-term health risks associated with CRS. We also performed the risk analysis of cancer events according to the presence of comorbidities (Table 4). We detected that there was a significant increase in developing all types of cancer events regardless of the presence of comorbidities. However, the risk was higher in patients with CRS and comorbidities (HR = 1.20, 95% CI = 1.02–1.43) than in patients with CRS without comorbidities (HR = 1.14, 95% CI = 1.01–1.28).

### 3.4. Risk Analysis of Overall Cancer Events According to Chronic Rhinosinusitis Phenotype

To gain a deeper understanding of the relationship between CRS and cancer risk, we analyzed cancer events according to different CRS phenotypes, as outlined in Table 5. Our analysis revealed a striking difference between the two CRS phenotypes in terms of cancer risk. Patients with CRSsNP were found to have a significantly elevated risk of all types of cancer compared to individuals without CRS, suggesting that the absence of nasal polyps in CRS may be a key factor contributing to this heightened cancer risk. In contrast, no significant association was observed between CRS and cancer risk in patients with CRSwNP. This lack of a clear link in the CRSwNP group suggests that the presence of nasal polyps may alter the pathway through which CRS influences cancer development or that other factors associated with CRSwNP may mitigate cancer risk. These findings highlight the potential importance of CRS phenotype as a factor in assessing cancer risk in patients with CRS and suggest that CRSsNP may be a particular phenotype of concern for clinicians when considering long-term cancer surveillance strategies. Further studies are required to explore the underlying mechanisms that may explain the different cancer risks associated with the two CRS phenotypes.

## 4. Discussion

Chronic inflammation is associated, both directly and indirectly, with a wide range of long-term health conditions, including atherosclerosis, myocardial infarction, chronic heart failure, Parkinson’s disease, Alzheimer’s disease, asthma, diabetes, psoriasis, osteoporosis, and various forms of cancer [13,14,15]. To the best of our knowledge, this study is the first comprehensive evaluation of the risk of developing all types of cancer in a large cohort of patients diagnosed with CRS. This nationwide cohort study involved a robust sample of 10,567 patients with newly diagnosed CRS and 42,268 matched controls without the condition. In this study, patients with CRS exhibited a significantly higher risk of developing overall cancer events than the control group, underscoring the noteworthy association between CRS and cancer risk. The adjusted HR for cancer development in the CRS cohort was 1.16 (95% CI: 1.05–1.28), indicating that patients with CRS have a 16% higher risk of experiencing cancer events compared to individuals without CRS, after adjusting for potential confounders. Previous studies have shown that prolonged inflammation can lead to the activation of various signaling pathways involved in cell proliferation, survival, and DNA repair, all of which are critical in the development of cancer [16,17,18]. Additionally, chronic inflammation enhances pro-oncogenic activity with decreased function of tumor suppressor genes, causes genomic instability, and induces tumors by promoting tumor cell proliferation and survival [19,20,21]. Furthermore, chronic inflammation can lead to DNA damage, primarily through the action of reactive oxygen and nitrogen species, which can induce lesions in DNA bases and the DNA backbone, either directly or through reactive intermediates generated during lipid peroxidation [22,23]. Thus, this ongoing inflammatory process may not only alter the structure and function of the sinuses but may also have systemic effects, increasing susceptibility to cancer in other organs. Consequently, several studies have proposed a potential association between chronic inflammation in patients with CRS and an increased risk of developing cancer, particularly in the head and neck region [8,9,10,11,12]. Collectively, our findings highlight the potential long-term oncological implications of CRS and the need for increased surveillance of these patients.

Unlike previous studies, this study investigated differences in cancer risk according to sex and CRS phenotype. Specifically, we found that female patients with CRS had a notably higher risk of developing cancer, suggesting that women with CRS may be more susceptible to malignancy than their male counterparts. This observation indicates the possibility of sex-related differences in the mechanisms linking CRS to cancer risk, potentially due to hormonal, genetic, or immune-related factors that differ between men and women. Similar to this finding, previous studies have shown sex differences in disease severity in patients with CRS, suggesting that hormonal differences may contribute to sex differences in disease symptoms [24,25,26,27]. However, this study included more women than men, which may have contributed to the higher cancer risk in women than in men. Interestingly, our subgroup analysis also revealed that patients with CRSsNP had a significantly increased risk of cancer, highlighting the distinct association between this CRS phenotype and cancer incidence. In contrast, patients with CRSwNP did not show the same elevated risk of cancer, suggesting that the presence of nasal polyps may alter the underlying mechanisms that contribute to cancer risk in patients with CRS. The phenotypic expression of CRS is believed to be significantly influenced by the balance between Th1 and Th2 immune responses. This immunological dichotomy is critical for shaping the distinct clinical presentations and inflammatory profiles observed in patients with CRS. Numerous studies have provided valuable insight into this relationship. Specifically, CRSsNP is associated with the upregulation of Th1 cytokine expression, accompanied by a predominance of neutrophilic infiltration in the affected tissues. This Th1-dominant inflammatory pattern suggests a cellular immune response that may contribute to the clinical and pathological characteristics of CRSsNP [28,29,30,31]. Several studies have shown that CRSsNP patients, in whom Th1 immune response is the primary pathophysiology, are associated with the presence or development of other diseases related to Th1 immunity [32,33,34]. Th1 cytokines such as tumor necrosis factor alpha (TNF-α) and interferon gamma (IFN-γ) are a group of pro-inflammatory cytokines that help the body fight intracellular pathogens. TNF-α is a pro-inflammatory cytokine that plays a key role in the connection between chronic inflammation and cancer [35]. TNF-α is involved in all stages of tumor development, from the initial transformation of cells to metastasis [36]. IFN-γ is an inflammatory factor that can regulate the initiation and resolution of inflammation. Thus, chronic inflammation induced by IFN-γ can be involved in the development and progression of cancer [16]. For these reasons, this discrepancy between CRS phenotypes emphasizes the need for further investigation into the role of different CRS subtypes in influencing cancer risk and provides valuable insights for clinicians in terms of risk stratification and personalized monitoring strategies for patients with CRS.

Despite its significant findings and strengths, this study has some limitations that should be carefully considered when interpreting the results. First, the identification of specific diseases in this study was based on diagnostic codes rather than detailed medical records, such as comprehensive medical histories or pathologic reports. This approach carries the inherent risk of misclassification bias. Additionally, in this cohort study, CRSsNP is much more frequent than CRSwNP. This difference may be due to misclassification bias resulting from coding chronic rhinitis patients as CRSsNP, in addition to the difference in incidence. Second, the study lacked access to detailed information about the duration and severity of CRS, including commonly used measures such as the Lund-Mackay and Lund-Kennedy scores. This limitation makes it impossible to determine the effect of CRS duration or severity on the risk of cancer development. Therefore, important insights into how the chronicity or intensity of CRS symptoms influences the likelihood of cancer remain elusive. Third, we were unable to include data on the carcinogenic histology or tumor stage of the study subjects. Consequently, the potential effects of CRS on the development of specific types of cancer could not be assessed, leaving an important aspect of the CRS-cancer relationship unexplored. Moreover, it is also possible that this difference in risk is because patients with cancer may have a higher incidence of CRS than those without cancer. Fourth, the timing of sinus surgery in patients with CRS may have influenced the occurrence of cancer events. Some patients experienced relatively long intervals before undergoing sinus surgery, whereas others experienced shorter intervals. This variability in the timing of the surgical intervention could potentially affect the observed associations and remains an area that requires further investigation. Fifth, the dataset used in this study did not include critical health-related variables, such as body mass index, lipid profiles, or information on behavioral risk factors, including smoking habits and alcohol consumption. The absence of these factors meant that we could not control for possible confounding effects on the observed relationship between CRS and cancer risk, which potentially limits the comprehensiveness of our findings. Finally, the retrospective design of this study restricted our ability to directly investigate and analyze the underlying pathological mechanisms that link CRS and cancer. Without prospective data, we cannot explore the causative pathways or biological interactions in depth, leaving these important mechanisms speculative rather than definitive.

## 5. Conclusions

Our study revealed that CRS is associated with an elevated risk of all types of cancer. Notably, female patients with CRS and patients with CRSsNP demonstrated a particularly high risk of cancer. These results emphasize the importance of vigilant monitoring and implementation of personalized treatment strategies for patients with CRS, focusing on those at greater risk for malignancy. These findings underscore the complex relationship between CRS, its subtypes, sex, and cancer risk, calling for further research to unravel the underlying biological mechanisms and inform clinical practices aimed at reducing cancer risk in patients with CRS.

## Figures and Tables

**Table 1:** Characteristics of the study participants.

Variables	Control (*n* = 42,268)	CRS (*n* = 10,567)	*p*-Value
Sex			0.9
Male	18,680 (44%)	4670 (44%)	
Female	23,588 (56%)	5897 (56%)	
Ages			0.9
15–39	24,416 (58%)	6104 (58%)	
40–64	15,352 (36%)	3838 (36%)	
>64	2500 (5.9%)	625 (5.9%)	
Residence			0.9
Seoul	11,404 (27%)	2851 (27%)	
Second area	11,480 (27%)	2870 (27%)	
Third area	19,384 (46%)	4846 (46%)	
Household income			0.9
Low (0–30%)	7212 (17%)	1803 (17%)	
Middle (30–70%)	10,256 (24%)	2564 (24%)	
High (70–100%)	24,800 (59%)	6200 (59%)	
Comorbidity			0.9
No	35,660 (84%)	8915 (84%)	
Yes	6608 (26%)	1652 (26%)	

CRS: chronic rhinosinusitis; three residential regions (Seoul, the largest metropolitan area in South Korea; other metropolitan cities; and small cities and rural areas); household income (low [≤30.0% of the national median], middle [30.1–69.9% of the national median], and high [≥70.0% of the national median]).

**Table 2:** The incidence and risk of overall cancer between the control and CRS groups.

Variables	N	Case	Person-Years	Incidence	Unadjusted HR (95% CI)	Adjusted HR (95% CI)
Overall Cancer						
Control	42,268	1817	12,382	0.147	1.00 (ref)	1.00 (ref)
CRS	10,567	547	3279.1	0.167	1.26 (1.15–1.39)	1.16 (1.05–1.28)

CRS: chronic rhinosinusitis; HR: hazard ratio; CI: confidence interval.

**Table 3:** Hazard ratio of incident overall cancer events by sex between the comparison and CRS groups.

Sex	Male		Female	
	Comparison	CRS	Comparison	CRS
Unadjusted HR (95% CI)	1.00 (ref)	1.19 (1.02–1.38)	1.00 (ref)	1.33 (1.17–1.50)
Adjusted HR (95% CI)	1.00 (ref)	1.50 (0.90–1.22)	1.00 (ref)	1.24 (1.10–1.41)

CRS: chronic rhinosinusitis; HR: hazard ratio; CI: confidence interval.

**Table 4:** Hazard ratio of incident overall cancer events by comorbidities between the comparison and CRS groups.

Comorbidities	With Comorbidities		Without Comorbidities	
	Comparison	CRS	Comparison	CRS
Unadjusted HR (95% CI)	1.00 (ref)	1.30 (1.10–1.54)	1.00 (ref)	1.21 (1.07–1.36)
Adjusted HR (95% CI)	1.00 (ref)	1.20 (1.02–1.43)	1.00 (ref)	1.14 (1.01–1.28)

CRS: chronic rhinosinusitis; HR: hazard ratio; CI: confidence interval.

**Table 5:** The incidence and risk of incident overall cancer events according to the CRS subtype.

Variables	N	Case	Person-Year	Incidence	Unadjusted HR (95% CI)	Adjusted HR (95% CI)
Comparison	42,268	1817	12,382	0.147	1.00 (ref)	1.00 (ref)
CRSsNP	10,417	538	3236.8	0.166	1.26 (1.14–1.39)	1.16 (1.05–1.28)
CRSwNP	1510	9	42.3	0.213	1.50 (1.07–2.90)	1.23 (0.64–2.36)

CRS: chronic rhinosinusitis; CRSsNP: chronic rhinosinusitis without nasal polyps; CRSwNP: chronic rhinosinusitis with nasal polyps; HR: hazard ratio; CI: confidence interval.

## Data Availability

All pertinent data supporting the conclusions of this study are provided in the analysis. Additional data may be requested for further review, with access contingent on institutional approval and data protection guidelines.

## Abbreviations

The following abbreviations are used in this manuscript:

CI	confidence interval
CRS	chronic rhinosinusitis
CRSsNP	chronic rhinosinusitis without nasal polyposis
CRSwNP	chronic rhinosinusitis with nasal polyposis
HR	hazard ratio
ICD	International Classification of Diseases
NHIS	National Health Insurance Service
